# Interhemispheric interplay between the left and right premotor cortex during grasping as assessed by dynamic causal modelling

**DOI:** 10.1038/s41598-023-31602-y

**Published:** 2023-03-27

**Authors:** Federica Bencivenga, Maria Giulia Tullo, Valentina Sulpizio, Gaspare Galati

**Affiliations:** 1 PhD Program in Behavioral Neuroscience, Department of Psychology, “Sapienza” University of Rome, Rome, Italy; 2 Brain Imaging Laboratory, Department of Psychology, “Sapienza” University of Rome, Via Dei Marsi, 78, 00185 Rome, Italy; 3 Cognitive and Motor Rehabilitation and Neuroimaging Unit, Santa Lucia Foundation (IRCCS Fondazione Santa Lucia), Rome, Italy; 4 Department of Translational and Precision Medicine, “Sapienza” University of Rome, Rome, Italy

**Keywords:** Motor control, Neural circuits

## Abstract

Research on the contribution of the ipsilateral hemisphere to unilateral movements, and how it is mediated by transcallosal connections, has so far provided contradictory findings. By using dynamic causal modelling (DCM) and Parametric Empirical Bayes analyses applied to fMRI data, we sought to describe effective connectivity during pantomimed and imagined right-hand grasping within the grasping network, namely the anterior intraparietal sulcus, ventral and dorsal (PMd) premotor cortex, supplementary motor area and primary motor cortex (M1). The two-fold aim of the present work was to explore a) whether right and left parieto-frontal areas show similar connectivity couplings, and b) the interhemispheric dynamics between these regions across the two hemispheres. We detected a network architecture comparable across hemispheres during executed but not imagined grasping movements. Furthermore, during pantomimed grasping the interhemispheric crosstalk was mainly driven by premotor areas: we found an inhibitory influence from the right PMd toward the left premotor and motor areas and excitatory couplings between homologous ventral premotor and supplementary motor regions. Overall, our results support the view that dissociable components of unilateral grasping execution are encoded by a non-lateralized set of brain areas complexly intertwined by interhemispheric dynamics, whereas motor imagery obeys different principles.

## Introduction

Despite the major recruitment of the contralateral hemisphere, the ipsilateral motor and premotor cortex participate in refining the execution of unilateral movements. Interhemispheric dynamics driving such contributions are mediated by transcallosal fibers in the corpus callosum^1^ and are supposed to act bidirectionally. The underlying neurophysiological mechanisms are known as interhemispheric inhibition (IHI) and facilitation (IHF) and in humans they can be directly tested using transcranial magnetic stimulation (TMS). Pioneer studies on these mechanisms have described IHI as the inhibition of the motor cortex ipsilateral to the moving hand exerted by the contralateral motor^2^ and premotor^3^ cortex. During unilateral movements, such a mechanism is functional to prevent mirror motor command of the contralateral body part which must be kept still^4^. These findings have challenged the contralateral control tenet, namely the assumption that each hemisphere controls the contralateral portion of the body exclusively based on inputs from motor and sensory crossing fibres.

While this evidence is roughly straightforward for simple movements, more complex movements have been proved to rely on a more bilateral involvement of both motor and premotor areas, which may likely require a different interhemispheric balance with respect to the above-described one. Multiple evidence points toward this view in the case of grasping movement, whose planning and execution depend on the bilateral activity of the anterior intraparietal sulcus (aIPs), ventral (PMv) and dorsal (PMd) premotor cortex, and supplementary motor area (SMA). TMS studies have shown that a bilateral virtual lesion of aIPs is necessary to impair hand shaping during grasping^5^. Similarly, lesions to both the left and right PMv lead to an impairment in motor planning during right-hand grasping, since this area bilaterally encodes hand posture. Differently, only the left PMv is responsible for the hand muscle recruitment hence being more directly involved in movement execution^6^. A body of literature using multivoxel pattern analysis (MVPA) applied to functional magnetic resonance imaging (fMRI) data has suggested that bilateral parietal and premotor regions encode limb-independent, high-level neural representations of the movement plan^7^ entailing the processing of temporal and spatial information both during motor execution and imagery^8,9^. An intriguing debate has been raised on the role of the ipsilateral motor cortex (iM1), whose activity is only marginally reported by fMRI activation studies. Davare and colleagues^5^ reported that interfering with the activity of iM1 during grasping alters the timing of muscle recruitment. fMRI studies have proved that activity in the ipsilateral motor cortex increases with movement complexity^10^; similarly, Verstynen and Ivry^11^ have shown that fluctuations in the activity of iM1 correlate with the activity in the contralateral motor cortex (cM1) and that this correlation is higher for complex movements. A possible explanation is that iM1 provides additional resources to correctly perform complex movements, coordinating the sequencing of muscle recruitment^5,12^.

Studying interhemispheric inhibitory and facilitatory mechanisms through fMRI is challenging since the blood oxygenation level dependent (BOLD) signal is much more influenced by excitatory rather than inhibitory spikes. A study conducted by Bestmann and colleagues^13^ sought to draw a broader picture of these phenomena by combining TMS and fMRI during a left-hand grip task. These authors found an inhibitory pattern at rest linking the left PMd with the right M1 and PMd, which turned to be facilitatory during the task. Together, this body of evidence strengthens the controversies surrounding different facets of interhemispheric motor dynamics, such as their directions and involved nodes, suggesting that they may not be univocal but rather task-dependent.

A more recent line of research is getting advantage of connectivity approaches to investigate the task-dependent interplay between brain areas. In this scenario, the analysis of effective connectivity performed through Dynamic Causal Modeling (DCM,^14^) may overcome fMRI limits by describing inhibitory and excitatory modulations between brain areas driven by experimental inputs. This approach has already been exploited to investigate interhemispheric couplings during hand movements. During a fist-closing movement, Grefkes and colleagues^15^ have shown that motor areas contralateral to the moving hand exert a suppressive influence on the ipsilateral motor areas. Gao and colleagues^16^ provided evidence of a modulation of the connectivity from the contralateral toward the ipsilateral SMA during execution and to less extent during imagery of a right-hand finger tapping movement. A study by Begliomini and colleagues^17^ focused on the interhemispheric couplings between homologous areas during grasping and reported modulation of the connectivity from the left aIPs to the right aIPs, and between the left and right PMd.

Additional insights on the contribution of the ipsilateral hemisphere to unilateral movements can be provided by comparing motor execution to motor imagery. These two processes recruit partially overlapping brain circuits dealing with action planning^18–21^. Although univariate fMRI analyses may have failed in detecting right-hemisphere activations for right-hand imagined movements, MVPA studies revealed cross-modal decoding in bilateral parietal areas^8,22^, supporting the view that such areas encode both abstract and concrete action properties^23^. This paves the way to the investigation of right-hemisphere dynamics during both executed and imagined actions, a matter being neglected by previous DCM studies^16,24^ that focused only on the contralateral hemisphere to the executed/imagined unilateral movements.

To tackle this issue, we used DCM and parametrical empirical Bayes (PEB^25^;). We adopted a similar procedure to that of our previous study^26^ in which we analysed left intrahemispheric couplings during a right-hand pantomimed grasping, and showed a serial involvement of aIPs, PMv, PMd, and M1, plus an additional role of SMA. Our previous findings^26^ supported the well-known contribution to grasping movement of each of these areas, being aIPs encoding the 3D representation of the object to be grasped, the PMv storing a “vocabulary” of grasp postures and goals, the PMd controlling finger configuration, and SMA controlling grip force scaling and sequence planning^27–29^, by providing additional evidence on the way these areas interplay during grasping movements. We also found that a similar, but less complex, motor program is planned during the imagination of the same movement. In the present study, using the same dataset we aimed at assessing the role of the homologous areas in the right hemisphere, as well as their interhemispheric dynamics. If the same processes are shared across hemispheres, the network should not only be bilaterally activated but also susceptible to interhemispheric crosstalk and similar intrahemispheric modulations despite movement lateralization. Conversely, if hemispheric-specific mechanisms occur, the bilateral activation of the network would be explained by different connectivity patterns. Furthermore, since movement execution and imagery tap into similar neural mechanisms, these statements should hold in both conditions, at least for higher-level processing areas such as the parietal regions.

To answer this challenging question, we conducted two separate DCMs: one exclusive for the right hemisphere, and the other leveraging intra-hemispheric results to estimate inter-hemispheric connections in a computational efficient fashion^30^. We hypothesized that we could observe in the right hemisphere a network architecture similar to the one we found in the left hemisphere^26^, confirming the existence of a bilateral network whose functions are shared across hemispheres; we also hypothesized that this bilateral circuit would be modulated by interhemispheric homologous and non-homologous bilateral couplings.

Our study stands out from the above-mentioned ones as we focused on a complex movement pantomimed execution and imagination, exploring the connectivity architecture within a wide bilateral network including parietal, premotor, and motor areas, finally investigating interhemispheric links between homologous and non-homologous brain regions. Furthermore, we exploited the hierarchical modeling implemented through PEB to run separate estimations of left and right couplings, whose empirical priors were subsequently used to estimate an interhemispheric model in a more robust and less computationally expensive way.

We provide evidence that unilateral pantomimed grasping, but not its imagination, elicits similar mechanisms in both hemispheres whose crosstalk is mediated by both excitatory and inhibitory influences.

## Methods

### Participants and tasks

In the present study, we reanalyzed BOLD data from a sample of twenty-five right-handed healthy subjects (22 females, mean age 26.5, s.d. 3.4) who participated in a previous study from our lab^21^ after giving their written informed consent. The study was approved by the local research ethics committee of the IRCCS Fondazione Santa Lucia in Rome, in accordance with the Declaration of Helsinki.

The fMRI exam consisted of the execution of a right-hand pantomimed grasping movement (“pantomimed grasping” condition) or its imagination (“imagined grasping” condition). The experiment was structured as a block design in which pantomimed and imagined grasping blocks were alternated with fixation blocks. Each block lasted 16 s and was introduced by a written instruction (1 s), followed by 8 trials. In each trial an object was shown in central vision for 300 ms, randomly chosen from a set of 36 black-and-white photographs of commonly used objects that elicited “whole hand” or “finger” grasping movements, followed by an interval of 1575 ms during which the subject had to perform or imagine the movement. Participants were instructed to imagine the object located in the proximity of their right hand, therefore excluding the transport component of the grasping movement. For further details on the protocol, see^21^.

### Image acquisition and analysis

MR images were acquired at the Neuroimaging Laboratory at Santa Lucia Foundation through a 3 T Siemens Allegra system. One structural image per subject was acquired through a Siemens MPRAGE sequence (TR = 2 s, TE = 4.38 ms, flip angle = 8°, 512 × 512 image matrix, 0.5 × 0.5 mm in-plane resolution, 176 contiguous 1 mm thick sagittal slices) and processed using FreeSurfer 5.1 (http://surfer.nmr.mgh.harvard.edu/) to obtain a surface representation of the individual cortex in a standard space, which was then transformed in the FS-LR space^31^ with 74 k nodes per hemisphere through the Connectome Workbench software (https://www.humanconnectome.org/software/get-connectome- workbench).

Two functional image time series per subject, each including 8 pantomimed grasping, 8 imagined grasping, and 4 fixation blocks, were acquired using a gradient-echo EPI sequence (TR = 2 s, TE = 30 ms, flip angle = 70°, 64 × 64 image matrix, 3 × 3 mm in-plane resolution, 30 slices, 2.5 mm slice thickness with no gap, ascending excitation order), corrected for head movements and coregistered to the structural images using SPM12 (Wellcome Department of Cognitive Neurology, London, UK), resampled to the individual surfaces using ribbon-constrained resampling as implemented in Connectome Workbench^32^, and smoothed along the surface with an iterative procedure emulating a Gaussian kernel with a 6 mm full width at half-maximum (FWHM).

Functional images were analyzed for each participant separately on a vertex-by-vertex basis, according to the general linear model (GLM), with execution and imagination blocks modeled as box-car functions convolved with a canonical hemodynamic response function, and framewise displacement (FD), a subject-specific time-series index of the overall estimate of movement over time^33^, used as a nuisance regressor. Group-level statistical parametric maps of the activation in each condition relative to the baseline (i.e., pantomimed grasping > fixation; imagined grasping > fixation t- contrasts) were obtained with a cluster-forming threshold of *p* < 0.001 and corrected for multiple comparisons at the cluster level (*p* < 0.05) through a topological false discovery rate procedure^34^.

### Region definition

We selected five regions of interest (ROIs) per hemisphere, namely the anterior intraparietal sulcus (aIPs), the ventral (PMv) and the dorsal (PMd) premotor cortex, the supplementary motor area (SMA), and the primary motor cortex (M1). ROIs were defined on the cortical surface reconstruction of each individual hemisphere as the regions responding stronger to the pantomimed grasping condition than the fixation (pantomimed grasping > fixation t-contrast). We applied a watershed segmentation algorithm^35^ on the statistical parametrical maps (*p* < 0.5) and selected single activation peaks and their neighborhood for a maximum of 300 cortical nodes. Finally, for each defined region and subject, we retained the first principal component (*eigenvariate*) of the adjusted timeseries to be entered in the DCM analysis. A regional analysis on the right-hemisphere ROIs was also performed to address the recruitment of each of these regions during motor imagery and pantomime.

### Dynamic causal modeling

We assessed right-hemisphere and interhemispheric dynamics during the execution and imagination of a pantomimed grasping through Dynamic Causal Modeling (DCM)^14^, implemented in SPM12. DCM estimates effective connectivity in terms of changes in neuronal states over time, by computing the intrinsic coupling among nodes (A matrix), the activation of the circuit exerted by a driven input (C matrix), and the modulatory effect of input on the connectivity between nodes (B matrix).

The interhemispheric model we aimed to test is composed of ten regions (5 per hemisphere), therefore requiring the estimation of a large number of parameters. In the DCM estimation, any model with more than 8 brain regions is estimated by setting some constraints on the model priors to avoid the potentially redundant parameterization of large DCMs. The built-in solution for that is to estimate the task-based functional connectivity, and then use its results to inform the priors of the effective connectivity analysis. Another valid procedure is suggested by Razi and colleagues (2017). Whenever a network can be meaningfully split into subnetworks, the authors propose to estimate effective connectivity in each subnetwork, and then use such results to estimate the whole network connectivity^30^.

Following this approach, we proceeded by conducting separate DCM-PEB analyses for each hemisphere and then used their results to inform the priors of a third, interhemispheric DCM. Note that the results for the left hemisphere have been discussed in a previous study^26^.

We built the connectivity model based on macaque tracer studies which offer the possibility to disambiguate the directionality of the fibers linking brain areas. The network architecture we tested here was the same we adopted for the left hemisphere in our previous work: in the A matrix (“baseline connectivity”), we included all the possible feedback and forward coupling among ROIs, except for the reciprocal connections between aIPs and the premotor and motor cortex (i.e., SMA, PMd, and M1) for which no anatomical evidence was provided by macaque tracer studies^36–50^ (see Supplementary Table 1 for the full list).

**Figure 1:**
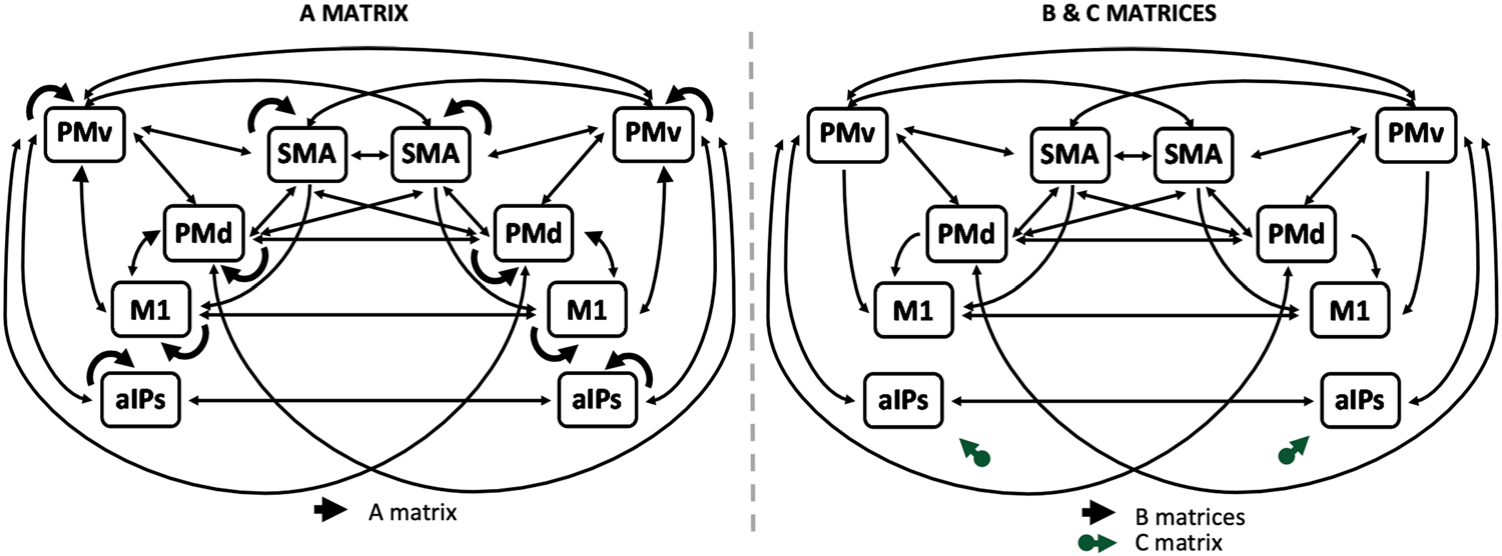
*Model for the effective connectivity analysis.* Black arrows represent connectivity between brain regions as modelled in the A (baseline; left panel) and B (modulatory; right panel) matrices. Modelled driven inputs (C matrix) are shown as green arrows in the right panel.

When testing the modulatory effect of the imagined and the pantomimed grasping on the effective connectivity between brain areas (B matrices), we investigated all the possible connections (forward and feedback) within the grasping network, but the ones from M1 to premotor areas. We modeled both pantomimed and imagined grasping conditions to exert a direct input only on aIPs (C matrix), as the entry hub of the grasping network devoted to the first processing stage for grasping movement, namely 3D encoding of visual stimuli. Figure 1 shows a graphical representation of the model architecture we tested.

Testing the same network architecture across hemispheres and conditions fulfilled the central scope of the present work, i.e., to address effective connectivity modulations in the grasping network based on the task (imagery or execution) and the hemispheric laterality (ipsilateral or contralateral to the moving hand). Note that, as we will discuss in the Results section, the “imagined grasping” condition yielded scarce whole-brain activations in the right hemisphere, but regional activations in the grasping network areas were significantly detected. We feel that this is an additional incentive to test whether a connectivity analysis could provide more insights than a massive univariate analysis in detecting the right-hemisphere contribution during right-hand grasping imagery.

Bayesian contrasts^51^ over the parameters that exceeded the threshold a) in both right and left DCMs for each condition, or b) within the right DCM in both conditions were implemented to evaluate differences between hemispheres and conditions.

Having estimated the two single-hemisphere DCMs, we built an interhemispheric model by using the group posterior estimates of the single-hemisphere DCMs to inform the priors for a new estimation^30^, building the model to keep only the parameters that exceeded the threshold in the single-hemisphere DCMs. In addition, we modeled interhemispheric bilateral couplings in line with anatomical evidence from macaque tracer studies, that supported the existence of bilateral interhemispheric connections between all the selected ROIs, except for the connections between aIPs and all the contralateral non-homologous regions (see Supplementary Table 1). Baseline (A matrix) connectivity was tested in all these connections. In the B matrices, we tested the modulation exerted by the two conditions on the bilateral connections between parietal and premotor areas, and the unilateral connections from premotor to motor areas, hence excluding connections from the right or left M1 toward the contralateral SMA, PMv, and PMd.

#### Estimation of DCM and parametrical empirical Bayes (PEB)

We specified and inverted the full DCMs for each individual using the Variational Laplace estimation scheme^52^. We next checked that all subjects had a good model estimation by controlling for the variance explained by the model. Subjects with a lower value than 10% were excluded from the group analysis, in agreement with standard practice for DCM^53^.

A parametric empirical Bayes (PEB^25,54^) approach was then used to determine group results through separate analyses for the A matrix, and the B and C ones. This analysis acts as a hierarchical model in which empirical priors at the group level could shrink the estimates at the individual level toward those that reach the maximum of the model evidence. Bayesian Model Reduction (BMR^54–56^) and Average (BMA^57,58^) were used to prune the connections with the least evidence and average the parameters. Finally, we evaluated whether a parameter contributed to the model evidence by retaining only those with strong evidence of being present vs absent, namely those with posterior probability > 0.95 accordingly to a threshold based on free energy.

## Results

Results are organized into four sections. We will first describe right-hemisphere whole-brain and regional activations evoked by pantomimed and imagined grasping movements. Then, we will focus on how the parietofrontal network entailing grasping actions is selectively modulated by grasping execution and imagery. In line with the separate analyses we performed, we will describe right-hemisphere couplings, compare them with the left-hemisphere ones, and then highlight the interhemispheric dynamics.

### Whole-brain and regional activations

**Figure 2:**
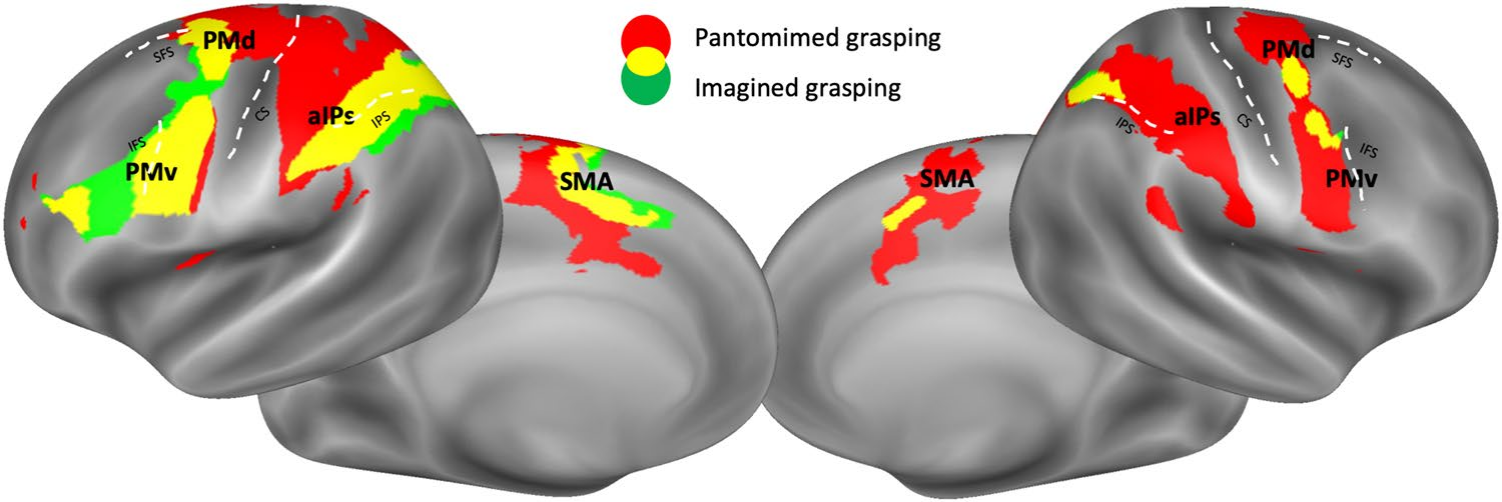
*Group whole-brain results.* Superimposition of the group activation maps resulting from the pantomimed grasping > fixation (in red) and the imagined grasping > fixation t-contrasts (in green) and average location of right hemisphere regions of interest (ROIs). Overlapping activated right brain regions across the two conditions are displayed in yellow. Maps are overlaid into an inflated Conte69 atlas^31^ of the right hemisphere. Average location of aIPs (anterior intraparietal sulcus area), PMv (ventral premotor area), PMd (dorsal premotor area), SMA (supplementary motor area), and M1 (primary motor cortex) is represented through black edges and labelled arrows. Anatomical landmarks are also reported (SFS: superior frontal sulcus; IFS: inferior frontal sulcus; CS: central sulcus; IPS: intraparietal sulcus).

During the pantomime of a grasping movement, activations were bilaterally detected in parietofrontal areas, including the intraparietal sulcus, premotor and motor areas, and prefrontal ones. Differently, motor imagery drove activation in the same parietofrontal network predominantly in the left hemisphere, as in the right one a subtle involvement of supplementary motor cortex, premotor cortex, and postero-occipital regions was detected (Fig. 2).

Five regions of interest (ROIs) were defined in each individual hemisphere based on the activation during the pantomimed grasping condition: anterior intraparietal sulcus area (aIPs), the ventral (PMv) and dorsal (PMd) premotor cortex, the supplementary motor area (SMA) and the primary motor cortex (M1).

**Figure 3:**
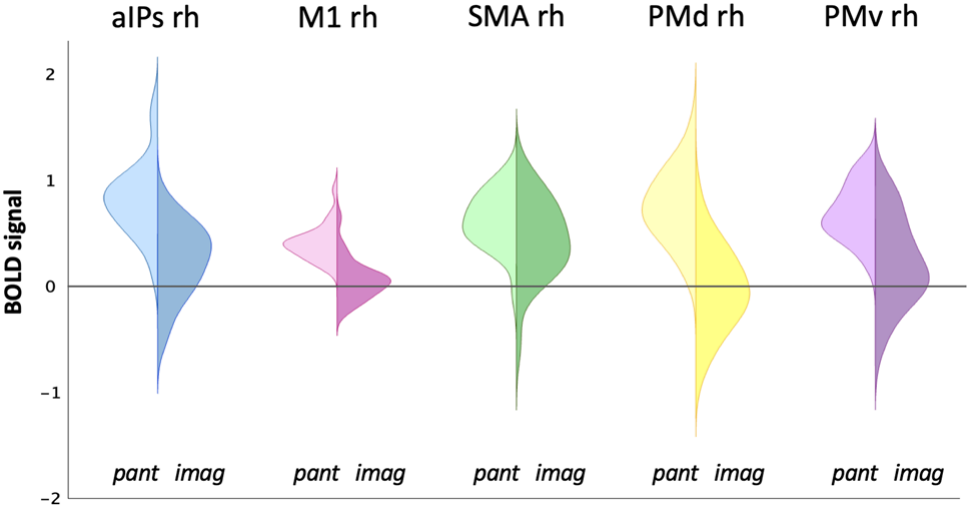
Plots of the activations of the right regions of interest (ROIs) in the pantomimed grasping and imagined grasping conditions. Violin plots showing the distribution of the activation of the selected ROIs across subjects are displayed for each right-hemisphere region, i.e., aIPs (blue), M1 (pink), SMA (green), PMd (yellow), and PMv (purple). The left side of the violins represents the pantomimed grasping condition (“pant”), and the right side the imagined grasping condition (“imag”).

A regional analysis was conducted to detect the recruitment of each ROI during the pantomimed and the imagined grasping condition, extending the results of the mass univariate group analysis. Results showed that all right-hemisphere ROIs were also significantly activated (*p* < 0.05 FDR corrected for the number of regions) during grasping imagery (Fig. 3; see Supplementary Fig. 1 in^26^ for similar results in the left hemisphere) with the following statistical values: aIPs rh (t_24_ = 3.66, *p* = 0.001), M1 rh (t_24_ = 3.18, *p* = 0.001), SMA rh (t_24_ = 3.57, *p* = 0.002), PMv rh (t_24_ = 2.28, *p* = 0.017) and PMd rh (t_24_ = 1.77, *p* = 0.042).

### Right-hemisphere DCM-PEB results

22 subjects out of 25 reached an explained model variance higher than 10% and were included in the PEB analysis. Results of the A matrix representing the baseline connectivity pattern are shown in Supplementary Fig. 1.

The core of our work was to describe how pantomimed or imagined movements perturb parietofrontal connectivity, as described by the B and C matrices of the DCM analysis.

**Figure 4:**
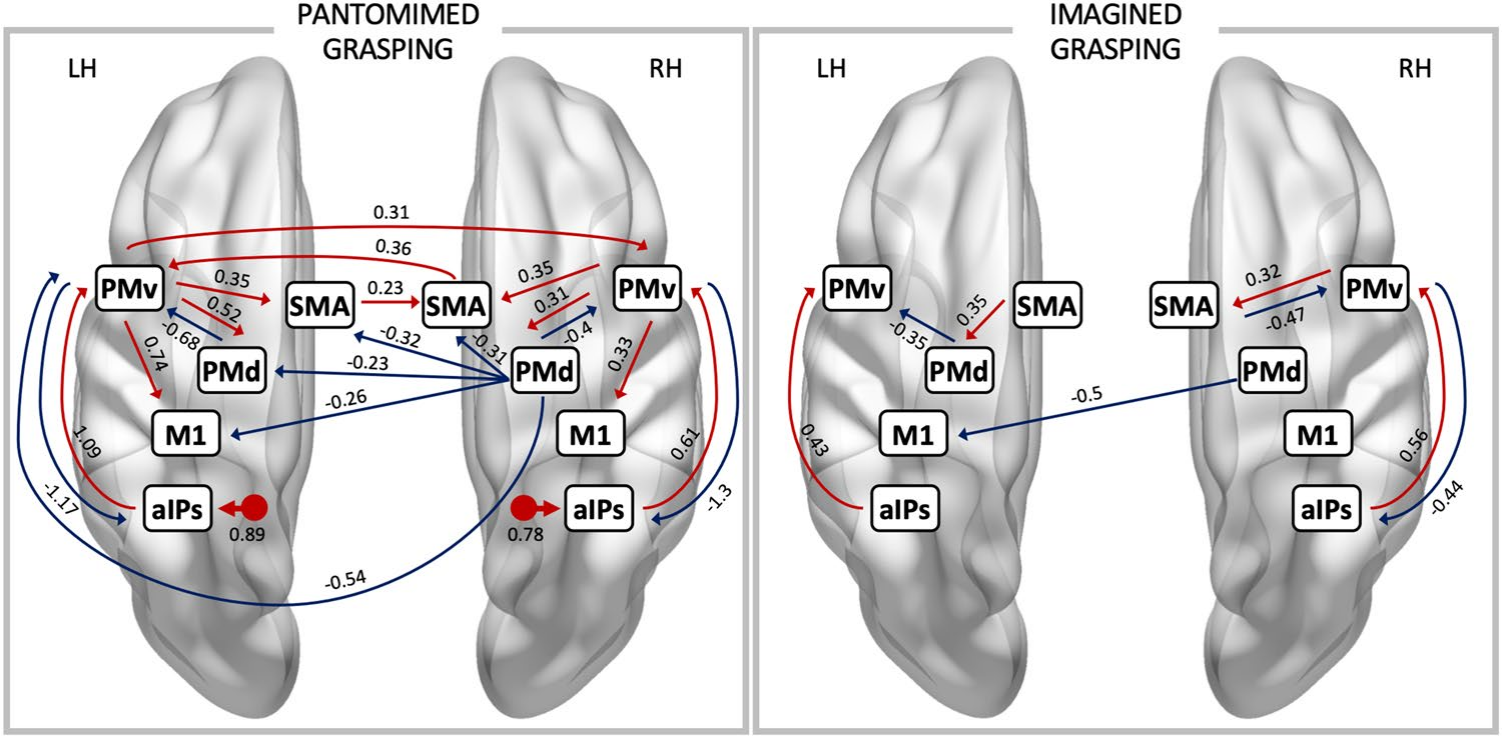
*PEB results—(B) and (C) matrices.* Schematic representation of the direct (**C** matrix) and modulatory (**B** matrix) effect on the effective connectivity within the network, separately for the pantomimed grasping (left panel) and the imagined grasping (right panel) condition. Only suprathreshold parameters (posterior probability > 0.95) are shown: red lines denote excitatory connections; blue lines stand for inhibitory connections. The direct effect (**C** matrix) on aIPs, where exceeding the threshold, is displayed as a dot with a left/right arrow. Connection strengths are also reported. Regions are denoted as aIPs (anterior intraparietal sulcus area), PMv (ventral premotor area), PMd (dorsal premotor area), SMA (supplementary motor area), and M1 (primary motor cortex).

The “pantomimed grasping” condition exerted a direct effect on the right aIPs (C matrix). Results of the B “modulatory” matrix showed a strong positive modulatory effect propagating from aIPs to PMv, followed by inhibitory feedback exerted by PMv toward aIPs. This is evidence of a feed-forward parietofrontal loop subserving grasping movements. PMv exerted a positive influence on M1, PMd and SMA. In turn, only PMd had a negative influence on PMv. PMd inhibited SMA (Fig. 4, left panel).

In the “imagined grasping” condition, the driven effect on aIPs (C matrix) did not exceed the threshold. In the B “modulatory” matrix, we found a positive influence from aIPs to PMv, and a feedback inhibition from PMv to aIPs. PMv and SMA were linked by excitatory/inhibitory connections (Fig. 4, right panel).

Across conditions, no differences were found in the aIPs to PMv (“Pantomimed grasping” = 0.61, “Imagined grasping” = 0.56, Posterior probability [pp] of the Bayesian contrast = 0.61) and in the PMv to SMA (“Pantomimed grasping” = 0.35, “Imagined grasping” = 0.32, Pp = 0.58) connections.

### Comparison between left- and right-hemisphere DCM-PEB results

**Figure 5:**
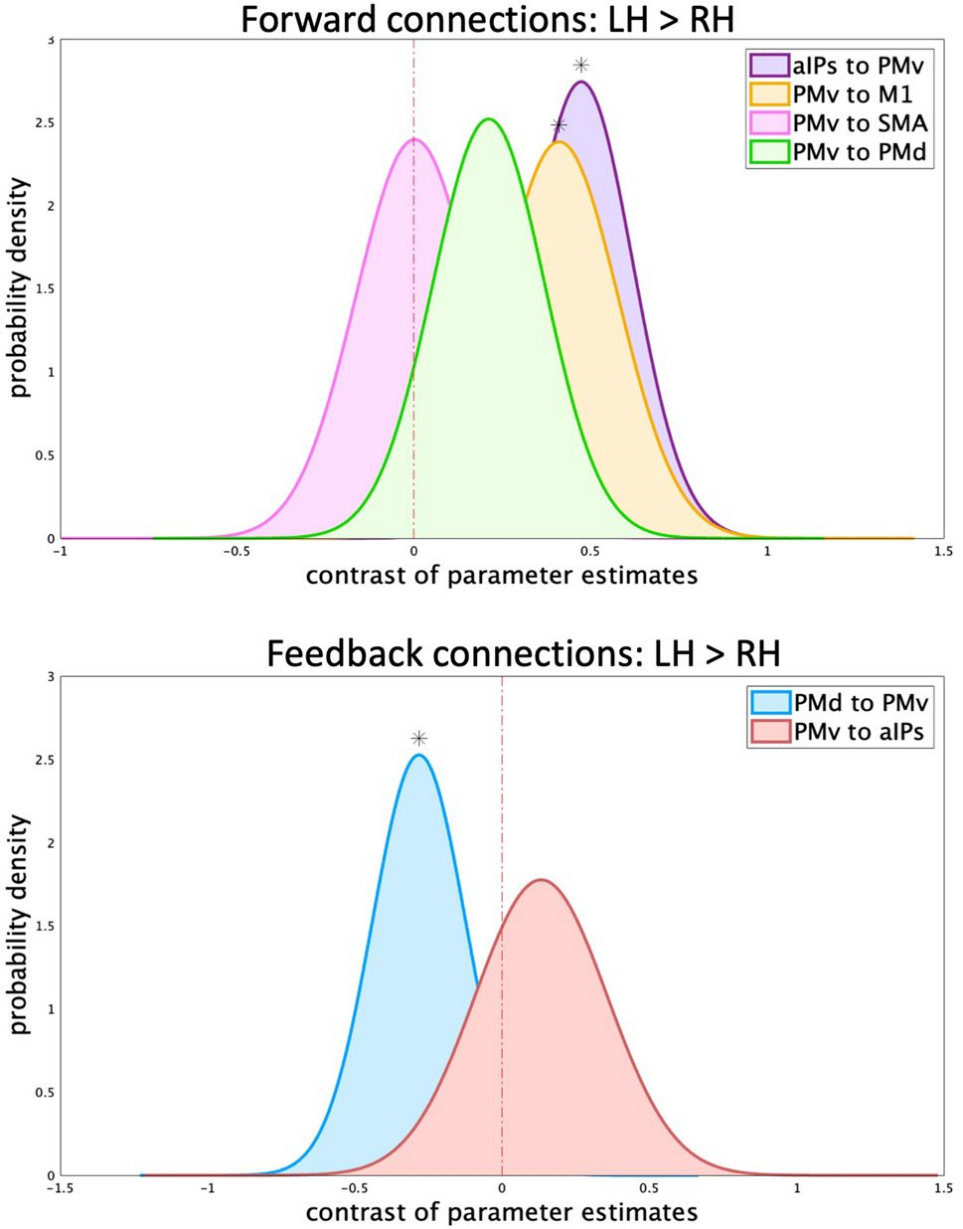
*Bayesian contrast over parameter estimates (LH* > *RH) in the “**pantomimed grasping**” condition.* Plot of the probability density function of the contrast over parameter estimates that exceeded the threshold (posterior probability > 0.95) representing the same connection in both single-hemisphere DCM. Connections are subdivided into Forward (upper panel: PMv to SMA in pink, PMv to PMd in green, PMv to M1 in yellow, aIPs to PMv in purple) and Feedback (lower panel: PMd to PMv in blue, PMv to aIPs in red). A red dotted line denotes the 0 point. Contrasts whose posterior is higher than 0.95 are marked with an asterisk above the probability density curve. If the curve is on the right (i.e., mean higher than 0) the connection is higher in the left vs the right hemisphere; conversely, if the curve is on the left (i.e., mean lower than 0) the connection has lower connection strength in the left vs the right hemisphere.

In the present section, we will compare the results of the right hemisphere DCM (B and C matrices) with the left hemisphere ones presented in our previous work^26^. We will also describe the suprathreshold (Posterior Probability > 0.95) Bayesian contrasts aimed to compare connection strengths of common modulated connections across the two hemispheres (Fig. 5).

As first striking evidence, connectivity patterns were extremely similar across hemispheres during pantomimed movements, but not their imagination.

In the “pantomimed grasping” condition, the positive forward connections from aIPs to PMv and PMv to M1 were stronger in the left than the right hemisphere (aIPs to PMv: LH = 1.09, RH = 0.61, Pp = 0.99; PMv to M1: LH = 0.74, RH = 0.33, Pp = 0.99). This is compatible with the predominance of left-hemisphere couplings for right-hand movements. The other forward connections (PMv to PMd and SMA) were not significantly different across hemispheres. The only feedback inhibitory connection differing across hemispheres was PMd to PMv (LH = − 0.68, RH = − 0.4, Pp = 0.96). Note that the connection from PMd to SMA was modulated in the right, but not the left hemisphere.

In the “imagined grasping” condition, only the positive influence from aIPs to PMv was common across hemispheres, without significant differences between left/right connection strengths (LH = 0.43, RH = 0.56, Pp = 0.83). This bets against the predominance of left-hemisphere couplings for right-hand imagined movements. Positive modulation from SMA to PMd and negative modulation from PMd to PMv were exclusively found in the left hemisphere.

### Interhemispheric DCM-PEB results

In the interhemispheric DCM model, 23 out of 25 subjects reached an explained model variance higher than 10%, being therefore included in the PEB analysis.

In the “pantomimed grasping” condition, the majority of interhemispheric connections were inhibitory: negative inputs were exerted from the right PMd toward the left primary motor (M1) and premotor (PMv, PMd, SMA) cortex. Noteworthy, the left SMA and PMv exerted a positive modulation of their right homologue areas, whereas the right SMA contributed to increasing activation in the left PMv (Fig. 4, left panel).

Only an inhibitory modulation exerted from the right PMd toward the left M1 was detected in the “imagined grasping” condition (Fig. 4, right panel). Such inhibition was stronger than in the “pantomimed grasping” condition, likely accounting for the suppression of the motor output (Posterior estimate “Pantomimed grasping” = − 0.26; Posterior estimate “Imagined grasping” = − 0.5; Pantomimed grasping > Imagined grasping Pp = 0.97).

## Discussion

“Contralateral control” is the core tenet of motor functioning, supported by the anatomical arrangement of motor and sensory fibers. Notwithstanding, multiple pieces of evidence agree that this is only half the story, as the contribution of the ipsilateral hemisphere holds a fundamental role in successful motor planning and execution. The advent of more sophisticated methods to assess connectivity within and between hemispheres, such as new TMS protocols and fMRI data analysis techniques, has boosted knowledge on the topic.

While TMS is an ideal candidate to infer the causal impact of one brain region on an interconnected area and ensures high temporal resolution, it can hardly deal with the complexity of a network consisting of multiple hubs that exert a reciprocal influence. Indeed, the majority of studies using TMS to assess interhemispheric dynamics have focused on the activity of M1, as the increase or decrease in amplitude of the motor-evoked potential (MEP) recorded through electromyography has been used as a hallmark of neural inhibition or facilitation.

DCM applied to fMRI data can be a valid method to detect complex interactions within a wider network and to assess directional influences between brain areas besides M1. Since caution is necessary when relating BOLD signal increases or decreases to actual neural states, we will discuss our results in light of those deriving from both techniques, being aware that it is not possible to find strict homologies.

As we evaluated the effective connectivity within the grasping network during the pantomime of right-hand grasping movements and their imagination, we will discuss our results separately for each experimental condition.

### Pantomimed grasping

The first evidence drawn from the “pantomimed grasping” condition pointed toward striking similar network connectivity across hemispheres: the dynamic interactions occurring between right parieto-frontal areas were similar to those of the left hemisphere, even though connection strengths were overall lower, a result highly consistent with that found by Begliomini and colleagues^17^. The signal spanned from aIPs to PMv, was susceptible to modulations by PMd and SMA, and finally reached M1. Notably, only the forward excitatory connections from aIPs to PMv and from PMv to M1 were stronger in the left vs the right hemisphere.

Beyond confirming the wide involvement of a bilateral parieto-frontal network during grasping, our results suggest that very similar mechanisms occur across hemispheres in terms of network functionality. As we already discussed in our previous work relative to the left-hemisphere results^26^, we suggest that a general motor program for grasping is planned by the aIPs-PMv circuit, whereas PMd and SMA encode high-level features of the movement. Notably, the excitatory input from the right PMv to the right M1 supports the view that the ipsilateral M1 provides additional resources when performing unilateral complex movements even if the amount of activation detected by fMRI is extremely low. Together, these findings support the key role of the ipsilateral hemisphere in the planning and execution of a complex unimanual action, whereby hand actions are supported by limb-invariant representations in parietal and frontal areas^7^.

The crucial issue we revamp here is that interhemispheric connections may bridge the two hemispheres to allow sharing of resources and information for a successful motor plan. For this reason, the second step of our work sought to describe interhemispheric dynamics occurring during the task. When comparing our results with previous findings, we are aware that the heterogeneity in the protocols, techniques, and tasks used to investigate interhemispheric dynamics may lead to apparently contradictory results. For instance, we observed a massive interhemispheric inhibition spanning from the right to the left hemisphere, whereas interhemispheric facilitation occurred in the opposite direction. Noteworthy, this latter pattern reverses the inhibition occurring at baseline (A matrix), therefore being a hallmark of the connectivity modifications depending on the current motor state, i.e., active or passive. While a short and a long latency interhemispheric inhibition emerges from contralateral PMd to M1 at rest according to TMS studies^59^, others have pointed out that interhemispheric dynamics change during the transition from a motor state to another, reflecting the switch from motor planning to implementation. For instance, Liuzzi and colleagues^60^, using a simple right-hand reaction time task, recorded a biphasic pattern of modulation exerted from the right PMd toward the left M1, namely early and late latency facilitation respectively evoked by movement selection and execution, while M1-M1 interactions were modulated only right before movement onset. With the current experimental design, we could not decompose grasping movements into planning and implementation phases, therefore future studies will be aimed at disentangling dynamic changes in interhemispheric connectivity according to each stage of the movement.

The role of the right PMd in driving the inhibition toward the left premotor and motor cortex deserves a spotlight. PMd covers a key role in decision making and is a central structure for the selection and initiation of voluntary actions^61,62^. In agreement with that, it has been suggested that PMd is responsible for the top-down processing of movement control both within and across hemispheres^63^. Beyond PMd, our data show that in the case of grasping movements, the left PMv is the central hub of the network as it groups inputs from the ipsilateral and the contralateral hemispheres.

Worthy of mention is the role of the left SMA which, together with the left PMv, exerts a positive influence on its right homologue. Notably, a similar modulation was found by Gao and colleagues^16^ during a finger tapping task, supporting the idea that the information transfer between bilateral SMAs plays a crucial role in both unimanual and bimanual movements^15,64,65^. Still, a different role of left and right SMA, independently of the effector (left or right hand), was suggested by White and colleagues^29^ in a TMS study on grip force. According to these authors, the left SMA is crucial for predicting the required grip force, overall encoding object dynamics, whereas the right SMA is more likely involved in the translation of object representation into motor commands. In line with this view, our results suggest that object dynamics encoded in the left SMA may converge in the right SMA, integrated with the generated motor command, and transferred back to the left PMv to drive the last stages of motor planning and execution.

TMS studies have consistently detected an interhemispheric inhibition between the two motor cortices, a result that we failed to replicate. Methodological and theoretical issues can account for this discrepancy. First, we did observe M1-M1 inhibition at rest, therefore our results must be interpreted by accounting for the evidence that this baseline inhibitory pattern is not strengthened or reduced during motor execution or imagery, in other words no task-dependent modulation emerged in the B matrix. Moreover, it has been suggested that iM1 shapes the muscular command in different ways depending on the stage of the motor execution, according to which this area can drive inhibitory or facilitatory inputs^66,67^ and shows unspecific activity for right- or left-hand movements during planning, which turns to be specific during execution^7^. Although here we could not account for this distinction, on a deeper analysis the comparison between present and previous findings suggests that, during simple movements, a strengthening of the cM1-iM1 crosstalk is necessary (see the DCM study by ^15^), whereas complex movements might rely upon a premotor rather than a direct motor interhemispheric modulation (see also^17^).

### Grasping imagery

When imagining the grasping movement, a different scenario emerged. A first striking difference uncovered by the whole-brain activation maps is the only subtle involvement of the right hemisphere during grasping imagery, a result that suggests a contralateral hemisphere dominance in motor imagery vs the bilateral involvement of the network during motor execution.

In line with that, only partial similarities in the effective connectivity across the two hemispheres were revealed by the DCM. Differently from what we described for the pantomimed grasping, the connection from aIPs to PMv was equally modulated in both hemispheres. Furthermore, SMA and PMd cooperated to inhibit PMv only in the left hemisphere. Conversely, in the right one a loop directly linking SMA and PMv emerged, as well as an inhibition from the right PMd to the left M1. Presumably, these dynamic interactions both drive motor imagery and concur to prevent excitation from the left PMv toward M1, resulting in the suppression of the motor output. This latter interpretation is in line with the concept of an “impulse-control” mechanism aimed at preventing overt activity in the right hand^68^.

Overall, these results point toward a different role of the right and left hemispheres during motor imagery. A previous study on motor imagery in stroke patients found that only right hemisphere damage impaired the timing estimation of imagined movements, sparing that of real movements^69^. These authors suggested that the right hemisphere is crucial for maintaining spatial information over time when internally simulating the motor pattern. As SMA is a key node in temporal processing ^70^, our results may be interpreted from the perspective that temporal encoding of the imagined movement may be processed by the right SMA and then this information is transferred to the right PMv to coordinate the imagined movement.

Together, we can conclude that motor execution and imagery share neural effector- and task-independent representations in high-order areas such as aIPs, whereas lower-order areas as SMA and PMd deem with effector- and task-dependent representation ^7,8,22^, a result confirmed by the different connectivity patterns we found across hemispheres and condition. This is in line with previous studies showing that motor planning triggers the recruitment of common areas to both motor execution and imagery ^18–21^.

Future studies may be designed with the specific aim to address the different functional contributions of the two hemispheres to motor imagery vs execution with compelling implications for rehabilitation practice, for instance guiding the choice of target areas for brain computer interfaces (BCI) protocols using MI on post-stroke patients.

## Conclusion

With the present study, we adopted a novel methodological approach to investigate interhemispheric dynamics during unilateral complex movements. Our results endorse the idea of a complex interplay both within and between hemispheres during grasping movements, whereby dissociable components of unilateral grasping are encoded by a non-lateralized set of brain areas entailing the abstract action representations ^23^, whereas the more concrete action representations may be task- and limb-dependent. As grasping execution directly targets bilateral aIPs (C matrix) and grasping imagery is subtended by different hemispheric contributions, we argue that the ipsilateral hemisphere signal does not merely reflect an efference copy of the contralateral motor command, but rather an active contribution to the refinement and implementation of the motor plan.

Our study is not exempt from limits. First, we used a pantomime movement, that only partially share neural representations with actual grasping including contact with the graspable object. Also, we focused on a set of ROIs to keep a reasonable number of nodes within the network, including areas within the dorsoventral visual stream well-known to be key areas in the grasping network.

Further studies on the interhemispheric dynamics are advisable to inform clinical research on the spread of information along the corpus callosum supporting complex motor functions in pathological conditions. Unwanted bilateral muscle activity for unilateral actions may be induced by lesional events directly damaging the corpus callosum, but also by aging, which incurs the degradation of callosal fibers ^71–73^. Recovery of impaired motor functions post-stroke is known to be supported by interhemispheric connectivity. A recent study showed that the contralesional aIPs supports grasping movements performed with the stroke-affected hand, overall suggesting that the contralesional hemisphere can reallocate resources to the ipsilesional one ^74^. The understanding of interhemispheric mechanisms may boost rehabilitation programs in the case in which age, strokes, traumatic brain injury, or temporary limb immobilization foster a re-balance of the bridges between hemispheres.

## Supplementary Information

Supplementary Information.

## Data Availability

Functional data used in the present study are available on Github (https://github.com/fbencive/graspingDCM_Data).

## References

[1] Zarei M (2006). Functional anatomy of interhemispheric cortical connections in the human brain. J. Anat..

[2] Ferbert A (1992). Interhemispheric inhibition of the human motor cortex. J. Physiol..

[3] Mochizuki H, Huang Y-Z, Rothwell JC (2004). Interhemispheric interaction between human dorsal premotor and contralateral primary motor cortex: Interhemispheric interaction of dorsal premotor area. J. Physiol..

[4] Mayston MJ, Harrison LM, Stephens JA (1999). A neurophysiological study of mirror movements in adults and children. Ann. Neurol..

[5] Davare M, Andres M, Clerget E, Thonnard J-L, Olivier E (2007). Temporal dissociation between hand shaping and grip force scaling in the anterior intraparietal area. J. Neurosci..

[6] Davare M (2006). Dissociating the role of ventral and dorsal premotor cortex in precision grasping. J. Neurosci..

[7] Gallivan JP, McLean DA, Flanagan JR, Culham JC (2013). Where one hand meets the other: Limb-specific and action-dependent movement plans decoded from preparatory signals in single human frontoparietal brain areas. J. Neurosci..

[8] Monaco S, Malfatti G, Culham JC, Cattaneo L, Turella L (2020). Decoding motor imagery and action planning in the early visual cortex: Overlapping but distinct neural mechanisms. Neuroimage.

[9] Papitto G, Friederici AD, Zaccarella E (2020). The topographical organization of motor processing: An ALE meta-analysis on six action domains and the relevance of Broca’s region. Neuroimage.

[10] Verstynen T, Diedrichsen J, Albert N, Aparicio P, Ivry RB (2005). Ipsilateral motor cortex activity during unimanual hand movements relates to task complexity. J. Neurophysiol..

[11] Verstynen T, Ivry RB (2011). Network dynamics mediating ipsilateral motor cortex activity during unimanual actions. J. Cogn. Neurosci..

[12] Yarosh CA, Hoffman DS, Strick PL (2004). Deficits in movements of the wrist ipsilateral to a stroke in hemiparetic subjects. J. Neurophysiol..

[13] Bestmann S (2008). Dorsal premotor cortex exerts state-dependent causal influences on activity in contralateral primary motor and dorsal premotor cortex. Cereb. Cortex.

[14] Friston KJ, Harrison L, Penny W (2003). Dynamic causal modelling. Neuroimage.

[15] Grefkes C, Eickhoff SB, Nowak DA, Dafotakis M, Fink GR (2008). Dynamic intra- and interhemispheric interactions during unilateral and bilateral hand movements assessed with fMRI and DCM. Neuroimage.

[16] Gao Q, Tao Z, Zhang M, Chen H (2014). Differential contribution of bilateral supplementary motor area to the effective connectivity networks induced by task conditions using dynamic causal modeling. Brain Connect..

[17] Begliomini C (2015). Exploring manual asymmetries during grasping: A dynamic causal modeling approach. Front. Psychol..

[18] Hashimoto R, Rothwell JC (1999). Dynamic changes in corticospinal excitability during motor imagery. Exp. Brain Res..

[19] Lorey B (2011). Activation of the parieto-premotor network is associated with vivid motor imagery—A parametric fMRI study. PLoS ONE.

[20] Munzert J, Lorey B, Zentgraf K (2009). Cognitive motor processes: The role of motor imagery in the study of motor representations. Brain Res. Rev..

[21] Sulpizio V (2020). Real and imagined grasping movements differently activate the human dorsomedial parietal cortex. Neuroscience.

[22] Zabicki A (2016). Imagined and executed actions in the human motor system testing neural similarity between execution and imagery of actions with a multivariate approach. Cereb. Cortex.

[23] Turella L, Rumiati R, Lingnau A (2020). Hierarchical action encoding within the human brain. Cereb. Cortex.

[24] Kasess CH (2008). The suppressive influence of SMA on M1 in motor imagery revealed by fMRI and dynamic causal modeling. Neuroimage.

[25] Friston K, Zeidman P, Litvak V (2015). Empirical bayes for DCM: A group inversion scheme. Front. Syst. Neurosci..

[26] Bencivenga F, Sulpizio V, Tullo MG, Galati G (2021). Assessing the effective connectivity of premotor areas during real vs imagined grasping: A DCM-PEB approach. Neuroimage.

[27] Castiello U, Begliomini C (2008). The cortical control of visually guided grasping. Neuroscientist.

[28] Gerbella M, Rozzi S, Rizzolatti G (2017). The extended object-grasping network. Exp. Brain Res..

[29] White O, Davare M, Andres M, Olivier E (2013). The role of left supplementary motor area in grip force scaling. PLoS ONE.

[30] Razi A (2017). Large-scale DCMs for resting-state fMRI. Netw. Neurosci. Camb. Mass.

[31] Van Essen DC, Glasser MF, Dierker DL, Harwell J, Coalson T (2012). Parcellations and hemispheric asymmetries of human cerebral cortex analyzed on surface-based atlases. Cereb. Cortex.

[32] Glasser MF (2013). The minimal preprocessing pipelines for the human connectome project. Neuroimage.

[33] Power JD, Barnes KA, Snyder AZ, Schlaggar BL, Petersen SE (2012). Spurious but systematic correlations in functional connectivity MRI networks arise from subject motion. Neuroimage.

[34] Chumbley J, Worsley K, Flandin G, Friston K (2010). Topological FDR for neuroimaging. Neuroimage.

[35] Mangan AP, Whitaker RT (1999). Partitioning 3D surface meshes using watershed segmentation. IEEE Trans. Vis. Comput. Graph..

[36] Jenny AB (1979). Commissural projections of the cortical hand motor area in monkeys. J. Comp. Neurol..

[37] Leichnetz GR (1986). Afferent and efferent connections of the dorsolateral precentral gyrus (area 4, hand/arm region) in the macaque monkey, with comparisons to area 8. J. Comp. Neurol..

[38] Matelli M, Camarda R, Glickstein M, Rizzolatti G (1986). Afferent and efferent projections of the inferior area 6 in the macaque monkey. J. Comp. Neurol..

[39] Stepniewska I, Preuss TM, Kaas JH (1993). Architectionis, somatotopic organization, and ipsilateral cortical connections of the primary motor area (M1) of owl monkeys. J. Comp. Neurol..

[40] Rouiller E (1994). Transcallosal connections of the distal forelimb representations of the primary and supplementary motor cortical areas in macaque monkeys. Exp. Brain Res..

[41] Boussaoud D, Tanné-Gariépy J, Wannier T, Rouiller EM (2005). Callosal connections of dorsal versus ventral premotor areas in the macaque monkey: A multiple retrograde tracing study. BMC Neurosci..

[42] Luppino G, Murata A, Govoni P, Matelli M (1999). Largely segregated parietofrontal connections linking rostral intraparietal cortex (areas AIP and VIP) and the ventral premotor cortex (areas F5 and F4). Exp. Brain Res..

[43] Luppino G, Matelli M, Camarda R, Rizzolatti G (1993). Corticocortical connections of area F3 (SMA-proper) and area F6 (pre-SMA) in the macaque monkey. J. Comp. Neurol..

[44] Geyer S, Matelli M, Luppino G, Zilles K (2000). Functional neuroanatomy of the primate isocortical motor system. Anat. Embryol. (Berl.).

[45] Marconi B, Genovesio A, Giannetti S, Molinari M, Caminiti R (2003). Callosal connections of dorso-lateral premotor cortex. Eur. J. Neurosci..

[46] Dum RP (2005). Frontal lobe inputs to the digit representations of the motor areas on the lateral surface of the hemisphere. J. Neurosci..

[47] Dancause N, Barbay S, Frost SB, Mahnken JD, Nudo RJ (2007). Interhemispheric connections of the ventral premotor cortex in a new world primate. J. Comp. Neurol..

[48] McGuire BA, Gilbert CD, Rivlin PK, Wiesel TN (1991). Targets of horizontal connections in macaque primary visual cortex. J. Comp. Neurol..

[49] Lanz F (2017). Distant heterotopic callosal connections to premotor cortex in non-human primates. Neuroscience.

[50] Ruddy KL, Leemans A, Carson RG (2017). Transcallosal connectivity of the human cortical motor network. Brain Struct. Funct..

[51] Dijkstra N, Zeidman P, Ondobaka S, van Gerven MAJ, Friston K (2017). Distinct top-down and bottom-up brain connectivity during visual perception and imagery. Sci. Rep..

[52] Friston K, Mattout J, Trujillo-Barreto N, Ashburner J, Penny W (2007). Variational free energy and the Laplace approximation. Neuroimage.

[53] Tak YW, Knights E, Henson R, Zeidman P (2021). Ageing and the ipsilateral M1 BOLD response: A connectivity study. Brain Sci..

[54] Friston KJ (2016). Bayesian model reduction and empirical Bayes for group (DCM) studies. Neuroimage.

[55] Friston K, Penny W (2011). Post hoc Bayesian model selection. Neuroimage.

[56] Pinotsis DA, Perry G, Litvak V, Singh KD, Friston KJ (2016). Intersubject variability and induced gamma in the visual cortex: DCM with empirical Bayes and neural fields: DCM With empirical Bayes and neural fields. Hum. Brain Mapp..

[57] Hoeting JA, Madigan D, Raftery AE, Volinsky CT (1999). Bayesian model averaging: A tutorial (with comments by M. Clyde, David Draper and E. I. George, and a rejoinder by the authors. Stat. Sci..

[58] Rosa MJ, Friston K, Penny W (2012). Post-hoc selection of dynamic causal models. J. Neurosci. Methods.

[59] Ni Z (2009). Two phases of interhemispheric inhibition between motor related cortical areas and the primary motor cortex in human. Cereb. Cortex.

[60] Liuzzi G, Horniss V, Zimerman M, Gerloff C, Hummel FC (2011). Coordination of uncoupled bimanual movements by strictly timed interhemispheric connectivity. J. Neurosci..

[61] Hoshi E, Tanji J (2007). Distinctions between dorsal and ventral premotor areas: Aanatomical connectivity and functional properties. Curr. Opin. Neurobiol..

[62] Cisek P, Kalaska JF (2005). Neural correlates of reaching decisions in dorsal premotor cortex: Specification of multiple direction choices and final selection of action. Neuron.

[63] Hinder MR, Fujiyama H, Summers JJ (2012). Premotor-motor interhemispheric inhibition is released during movement initiation in older but not young adults. PLoS ONE.

[64] Seitz RJ (2004). Bimanual recoupling by visual cueing in callosal disconnection. Neurocase.

[65] Stancak A (2003). Desynchronization of cortical rhythms following cutaneous stimulation: Effects of stimulus repetition and intensity, and of the size of corpus callosum. Clin. Neurophysiol..

[66] Murase N, Duque J, Mazzocchio R, Cohen LG (2004). Influence of interhemispheric interactions on motor function in chronic stroke. Ann. Neurol..

[67] Duque J (2005). Transcallosal inhibition in chronic subcortical stroke. Neuroimage.

[68] Gueugneau N (2013). Interhemispheric inhibition during mental actions of different complexity. PLoS ONE.

[69] Malouin F, Richards CL, Durand A (2012). Slowing of motor imagery after a right hemispheric stroke. Stroke Res. Treat..

[70] Coull JT, Charras P, Donadieu M, Droit-Volet S, Vidal F (2015). SMA Selectively codes the active accumulation of temporal, not spatial. Magnitude. J. Cogn. Neurosci..

[71] Baliz Y (2005). The influence of attention and age on the occurrence of mirror movements. J. Int. Neuropsychol. Soc..

[72] Bodwell JA, Mahurin RK, Waddle S, Price R, Cramer SC (2003). Age and features of movement influence motor overflow: Aging and motor overflow. J. Am. Geriatr. Soc..

[73] Hoy KE, Fitzgerald PB, Bradshaw JL, Armatas CA, Georgiou-Karistianis N (2004). Investigating the cortical origins of motor overflow. Brain Res. Rev..

[74] Hensel L (2022). Recovered grasping performance after stroke depends on interhemispheric frontoparietal connectivity. Brain.

