# Supplementary material for: Interhemispheric interplay between the left and right premotor cortex during grasping as assessed by dynamic causal modelling
Source: Sci Rep. 2023 Mar 27;13:4958. doi: 10.1038/s41598-023-31602-y (PMC10042845; doi:10.1038/s41598-023-31602-y)
Supplement: Supplementary file 1 — Supplementary Information. [file 41598_2023_31602_MOESM1_ESM.pdf]

Interhemispheric interplay between the left and right premotor cortex during grasping as assessed by Dynamic Causal Modeling

Federica Bencivenga, Maria Giulia Tullo, Valentina Sulpizio, and Gaspare Galati

To	aIPs.LH	M1.LH	SMA.LH	PMd.LH	PMv.LH	aIPs.RH	M1.RH	SMA.RH	PMd.RH	PMv.RH
**aIPs.LH**					Luppino 1999					
**M1.LH**			Luppino 1993<br>Dum & Strick 2005	Matelli 1986<br>Geyer 2000	Dum & Strick 2005		Jenny 1979<br>Leichnetz 1986<br>Rouiller 1994	Rouiller 1994	Rouiller 1994<br>Ruddy 2017	Dancause 2007
**SMA.LH**		Stepniewska 1993<br>Rouiller 1994		Stepniewska 1993<br>Luppino 1993	Stepniewska 1993<br>Luppino 1993		Rouiller 1994	McGuire 1991<br>Rouiller 1994	Rouiller<br>Ruddy 2017	Dancause 2007
**PMd.LH**		Matelli 1986<br>Dum & Strick 2005	Luppino 1993<br>Dum & Strick 2005		Matelli 1986<br>Marconi 2001		Marconi 2003<br>Boussaoud 1995<br>Lanz 2017	Marconi 2003<br>Lanz 2017	Marconi 2003<br>Lanz 2017	Marconi 2003<br>Dancause 2007<br>Lanz 2017
**PMv.LH**	Luppino 1999	Dum & Strick 2005	Dum & Strick 2005	Matelli 1986<br>Dum & Strick 2005			Dancause 2007<br>Lanz 2017	Dancause 2007<br>Lanz 2017	Dancause 2007<br>Lanz 2017	Boussaoud 1995<br>Dancause 2007<br>Lanz 2017
**aIPs.RH**										Luppino 1999
**M1.RH**		Jenny 1979<br>Leichnetz 1986<br>Rouiller 1994	Rouiller 1994	Rouiller 1994<br>Ruddy 2017	Dancause 2007			Luppino 1993<br>Dum & Strick 2005	Matelli 1986<br>Geyer 2000	Dum & Strick 2005
**SMA.RH**		Rouiller 1994	McGuire 1991<br>Rouiller 1994	Rouiller 1994<br>Ruddy 2017	Dancause 2007		Stepniewska 1993<br>Rouiller 1994		Stepniewska 1993<br>Luppino 1993	Stepniewska 1993<br>Luppino 1993
**PMd.RH**		Marconi 2003<br>Boussaoud 1995<br>Lanz 2017	Marconi 2003<br>Lanz 2017	Marconi 2003<br>Lanz 2017	Marconi 2003<br>Dancause 2007<br>Lanz 2017		Matelli 1986<br>Dum & Strick 2005	Luppino 1993<br>Dum & Strick 2005		Matelli 1986<br>Marconi 2001
**PMv.RH**		Dancause 2007<br>Lanz 2017	Dancause 2007<br>Lanz 2017	Dancause 2007<br>Lanz 2017	Boussaoud 1995<br>Dancause 2007<br>Lanz 2017	Luppino 1999	Dum & Strick 2005	Dum & Strick 2005	Matelli 1986<br>Dum & Strick 2005	

Supplementary Table 1. Anatomical macaque studies proving the existence of anatomical connections among regions of interest of the left and right hemisphere (aIPs, PMv, PMd, SMA and M1). Homologous regions (e.g., aIPs in the two hemispheres) are supposed to be reciprocally connected. Green cells represent existent anatomical connections; white cells stand for anatomical connections not reliably identified in macaques, or self-connections.

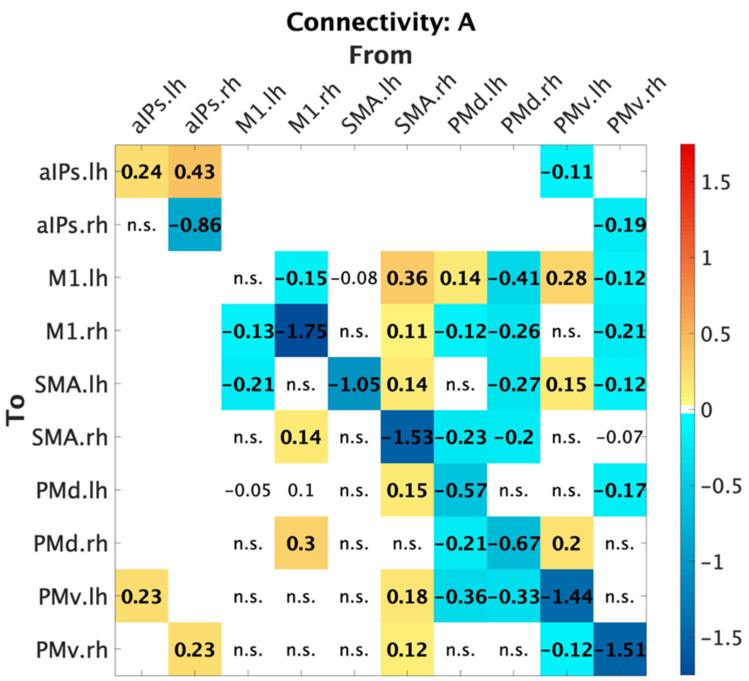

Supplementary Figure 1. *PEB results – A matrix.* Matrix of the effective connectivity of the unmodelled baseline; only suprathreshold parameters posterior probability > 0.95 are shown, whereas subthreshold parameters are marked with “n.s.” (i.e., non-suprathreshold), and non-modelled connections, i.e, whose priors are set to 0, are displayed in white. Connection strengths are represented in a scale from yellow to dark red, if excitatory, and from turquoise to dark blue, if inhibitory. Values of connection strengths are also provided.
